# Large Crown Cavities with Walls Unbroken

**Published:** 1872-10

**Authors:** E. L. Hunter


### ARTICLE IV.

### *Large Crown Cavities with Walls Unbroken.*

BY E. L. HUNTER, D. D. S.

In reading Dr. M. S. Dean's essay on "Sound Practice Sound Philosophy," I was struck with what appeared to me to be unsound practice and unsound philosophy. I may be in error; if I am, the ground work, the principles which I have been taught are erroneous, and I beg that those more enlightened will teach me.

The doctor says, "In filling *large* crown cavities with surrounding walls unbroken, the central portion should be kept higher than the peripheral, especially as the work approaches completion. If during the process of filling, the plug maintains a concave surface, the force applied tends to draw the gold away from the margins. These can be made perfect only by the principle of wedging. Gold should not be forced *unwillingly* into any portion of a filling where cohesion is important. The pellet and plug must unite by first intention, and before the gold has been rolled from place to place, and thus becoming partially condensed or knotted."

Now I ask, how will the doctor get wedge pressure with adhesive or cohesive pellets along the margin of said fillings, and at the same time get a union by the first intention? The very principle of a wedge is otherwise. If his filling is of cohesive, and none other is named, it must hold each pellet by cohesion; if of non-cohesive, and none other is adapted to the principle of wedging, it must all hold its position by contending pressures set up by wedging. It is very clear that small pellets may be forced down along a peripheral which is lower than the centre, and a good margin made provided the pellets are small enough, and the angle into which they are driven not too acute. Then, however, there will not be union by the first intention, and the pellet will keep its place both by wedging and cohesion, neither of which will be perfect. If the cohesion on the side of the pellet next the filling is sufficient to counterbalance the pressure of the perpendicular wall all well; if not, it will of course come away.

Our method of filling these cavities is to *keep* a slight concavity to the plug until the margins are completed by means of which we are enabled at all times to observe what adaptation we are making to the walls; we exert our force nearly always in the direction of the bottom of the cavity, or at least nearly parallel to the wall against, or touching which .s the pellet we are condensing.

Force upon the centre of a very poorly condensed filling does "have a tendency to draw it from the walls," but if the plug is condensed all the way, each pellet, from the bottom, any additional pressure *must expand* and *not contract* the gold. If great density has been had all along on its peripheral, the succeeding central pellets might be just as much a wedge as those the Dr. proposes to finish the margins with; with the additional advantage of cohering on all its sides. If there are any doubts of a sufficiently close union with the margins, a smooth point and mallet, when the cavity is just full, will settle all doubt. Again, if the peripheral, be kept higher than the centre, as we advance we have a ring next the margin being continually lengthened by its increasing density, and therefore pressing harder and harder against the walls. If the cavity was circular, and it would be generally somewhat so in the cavity named, the last pellet placed in the centre in the concavity of the plug, if drawing up under pressure, and there is no need of its doing so, could not draw in the margins, because the contractions would be against arches.

I have not written for the purpose of assailing in an unkind spirit, but that more information might be had on this subject, for in more intelligence in the details of making fillings must come benefit, if to you, you are welcome; if to me, so much the better.

